# Correspondence

**Published:** 1859-06

**Authors:** Abr. Robertson

**Affiliations:** Wheeling, Va.


### Correspondence.

WHEELING, Va., May 16, 1859.

MESSRS. EDITORS: In the last number of the *News Letter*, there are two articles *purporting* to be on the subject of "lateral pressure of the teeth"! and both these articles refer to a long paper read before the meeting of the American Dental convention and was published, I believe, in your journal of September last, and also in other leading Dental journals; and that long article refers to others *purporting* to be on the same subject.

I believe that the promulgation of error on any, and on all subjects tends to do harm; and on subjects as important as the health and comfort of mankind, is liable to do great harm.

I have looked over all the articles referred to, and, were it not for the *quasi*-endorsement they have received by being published in some of our leading and influential Dental journals—the senior editor of *one* of the journals I *know* to be a man of ability and scientific attainments—should have thought them, every one, so palpably erroneous that there could not be any danger of their misleading the most adolescent professional tyro. For, in all the cases cited and quoted, and they are quite numerous—I am not able to find the first indication of the state of things claimed, or the least particle of proof that there is any such thing as lateral pressure of the teeth. Do not anatomy, physiology and pathology all deny the possibility of any such thing? After the eruption of a tooth through the gum, and it has become fully developed, what power is there to produce lateral pressure? We are told by Dr. (?) McCalla, that there is "*a force from behind* exerted upon the advancing wisdom tooth"!! But what, oh learned *savan*, is that "force from behind"? There are no muscles in the gums to exert such force, or at least, as yet there have been none demonstrated. And if there were muscles there, how long would they continue to act, before they would accommodate themselves to the constant resistance? A few days at most. The alveolus has no elasticity to act as a spring upon the wisdom tooth to force it against other teeth after it has assumed its position. And if it were a spring, who bent and set it there, and for what purpose? And after it was set and bent, how long would it require for the other teeth to accommodate themselves to its pressure? What is this wonderful "force from behind"? How acts it?

I have brought the edge of an upper inscisor from within to without the arch of the lower inscisors, a full fourth of an inch, simply by the use of an inclined plane on which the tooth to be moved rested, when the child closed his mouth, and no compulsion used to make him close it, in four days, and the child scarcely make complaint of its being uncomfortable!

A very small piece of gum-elastic drawn between the two teeth, even when the arch is unbroken, will, in a single night, overcome all this "force from behind," and separate them half a line or so, making them, at worst, only a little sore; and if it is continued there a few days, all soreness, pain and uneasiness will be gone.

Every dentist who has given the attention to the regulating of irregular teeth that his *duty* requires, must know that by steady, though very light pressure, a tooth may be moved in any direction and almost to any extent, with nearly, if not quite, entire freedom from pain or soreness, and certainly with none of those neuralgic symptoms described in the articles alluded to; and I *suppose* if a wisdom tooth were to form in an entirely horizontal position, with its fangs in the ramus of the jaw, and the grinding surface of its crown directly against the posterior part of the fangs of the second molar, the process of formation is so slow, and the pressure would, therefore, be so slight and so uniform, that the parts would adapt themselves to their new relations without producing so much uneasiness even as to attract the attention of the patient. And I apprehend, too, that the same amount of pressure on a part designed and made to receive and to bear pressure like the teeth and peridental membranes, may be a somewhat different thing from the same pressure upon the gums as in the eruption of the teeth through them. But the simple truth is, that a row of ten-penny nails driven in an oak plank can just as easily crowd each other as a set of healthy teeth after they are fully developed. Of course, tumors of the gum or of the alveolus, or adventitious growth of their fangs, may produce lateral pressure upon the teeth; but that the natural growth or formation of the teeth can not produce any continued painful pressure upon the teeth, is very clearly shown by what has been already said of the effect of the rubber drawn between them, and more fully, still, by the fact that there is not room enough in the jaw for all the teeth to form evenly and perfectly, some of them will, unless art interferes, very quietly, but certainly, form without or within the proper arch, thus making irregularities. But we are told, by one of your correspondents, that where no other cause could be found to account for pain in the teeth and jaws, he *inferred* lateral pressure must be the cause, and proceeded to extract accordingly! and found, too, on getting out the teeth, that his diagnosis was correct *because the periosteum was in a state of inflammation, and the foramina of the teeth were enlarged and surrounded by sacs!* Now it happens, I believe, that all teeth, before they are fully developed, have large foramina and their membranes too, while engaged in secreting and depositing the elements of the teeth—that is, while forming the teeth—assume a higher degree of activity, which, *to uneducated* eyes, might appear to be inflammation; but that increased action, *apparent* inflammation, subsides in due time, and the foraminæ become small enough when the teeth are finished.

Dr. McCalla quotes a great many authorities to prove his vagary; among them, Professors Velpeau, Lymes, Tomes, Liston, and many other lesser lights, but unluckily for the doctor, not one of you, whose opinion is worth a penny-rush on anything, so much as intimates that there is any such thing as lateral pressure of the teeth! But all the cases which he quotes go to show that the eruption of the teeth, through the gums, and especially of the wisdom teeth, frequently produces inflammation and other serious lesions, and only that. It would have been just as appropriate—just as much to the point—for Dr. McCalla and his young friends to have quoted some essay read before some county medical society, by some young country doctor, on "infantile summer-complaint," all intended to prove that the alarming mortality of infants by diarrhea, during the hot season, is owing to the irritation produced by teething or the eruption of deciduous teeth through the gums! and he might, just with as much propriety, proceed to extract such teeth, and on the inference that there was lateral pressure. The analogy would be precisely the same, and the inference as just in the one case as in the other, and the trouble here would be found to depend just as much on "lateral pressure" here, as where we are told of having sacrifised teeth for that supposed cause; and the young gentlemen might have found the history of an alarming number of cases in any city, town, village or section of country in the nation, without the trouble of going to Scotland, England, and France.

But supposing Velpeau, Tomes, Lyme and Liston should concur in the opinion that neuralgia did frequently result from lateral pressure of the teeth, what influence would even their united opinion have on the mind of any man who had independently studied the anatomy of the teeth and their appendages, unless they could demonstrate the seat of that "force from behind"! And, great and learned as these men all professedly are, what weight would their opinions have with intelligent dentists anywhere, on matters pertaining to the teeth? The opinion of E. Parmley, Dunning or Foster of New York, of White or Arthur of Philadelphia, Harwood of Boston, or of Willard of New Hampshire, ought to outweigh them all. Velpeau, himself, says, in the introduction to the chapter on the diseases and operations on the teeth, in his great work on Surgery, edition edited by our own Mott, vol. i., p. 315,—"For this chapter I have not wished to rely on my own proper experience; I have desired my friend, Dr. Toirac, one of the most skillful and capable dentists of the capitol, to prepare it for me. The reader must, therefore, expect to find here the doctrines and precepts of this distinguished practitioner, rather than mine." In that chapter, p. 320, article on filling teeth, D. Toirac says, "We give this name to the operation by which we introduce lead into the cavities which the teeth present, in consequence of caries or particular alterations of the enamel. They formerly used, for this purpose, only sheet-lead in very thin laminæ; since that, recourse has been had to tin, which oxydises less; to gold, silver or platina leaf; and, finally, to the metal of Darcet, rendered more fusible by the addition of a sixteenth or twentieth of mercury; by means of the actual cautery, we melt this last composition in the cavity we wish to obliterate. The operation of filling is one of the most simple and easy." This chapter contains a large amount more, *equally interesting* and instructive, but which I can not now stop to quote.

Is not that splendid authority, at this day, to quote before what purports to be a scientific body of dentists, or to be published in our dental journals! Alas! alas! "who shall guard the shepherds."

If regular teeth so crowd each other as by pressure to produce pain—severe pain and suffering—what a set of "ninnies" I and you and the Tuckers and a thousand others, including the late eminent Townsend, have been, when, finding teeth so crowded as to force some of them out of their places, to go deliberately and apply extraneous power, and enough of it to overcome this "force from behind," and to compel them to assume their proper places! What a contest of opposing powers! and what a *tremendous lateral pressure* it must have made on the intervening teeth! Strange that it has not crushed their crisp crowns to crumbling atoms! Must not our patients have suffered intolerable torments by neuralgias, by necroses, by exfoliations, and—and—I know not what other ills? Who, after this, shall dare attempt to resist or counteract such a tremendous, though unseen force! Oh!

But, for argument's sake, let us admit that there is such a force as "lateral pressure of the teeth," and what shall we say of the treatment recommended, to-wit: the filing of spaces between them? Now, since the crowns of teeth are entirely insensible to any amount of pressure that will not crush them, if the wisdom teeth—from behind which this great force comes—should come at the same time and act with equal force, they would simply counteract each other, with scarcely a possibility of any harm; whereas, if a portion of the crowns were filed away, the necks of the teeth would impinge upon the gums and alveolus, and might produce some pain. And besides this, have not our young friends yet learned that when teeth are filed in that manner, that they are only a little time—longer or shorter—from destruction? Let the file, in all cases, be the last resort, I regard as a good general rule. But, aside from the risk of filing, forcing something between the crowns of the teeth, so as to insure an equal pressure, and to insure its being upon their crowns, would seem to me a far more reasonable treatment.

I did intend to make a few remarks on the effects sometimes produced by the eruption of the wisdom teeth, and of a want of space *between the jaws* for their full development; and upon the best means of removing them; but my paper has already become longer than I intended; I well, therefore, defer them till another number. ABR. ROBERTSON.

